# Context of action of Proline Dehydrogenase (ProDH) in the Hypersensitive Response of Arabidopsis

**DOI:** 10.1186/1471-2229-14-21

**Published:** 2014-01-13

**Authors:** Mariela Inés Monteoliva, Yanina Soledad Rizzi, Nicolás Miguel Cecchini, Mohammad-Reza Hajirezaei, María Elena Alvarez

**Affiliations:** 1 Centro de Investigaciones en Química Biológica de Córdoba CIQUIBIC, UNC-CONICET, Departamento de Química Biológica, Facultad de Ciencias Químicas, Universidad Nacional de Córdoba, Haya de la Torre y Medina Allende, Ciudad Universitaria, X5000HUA Córdoba, Argentina; 2 Leibniz Institute of Plant Genetics and Crop Plant Research (IPK), Molecular Plant Nutrition, Corrensstrasse 3, 06466 Gatersleben, Germany

**Keywords:** Proline metabolism, Proline dehydrogenase/oxidase, P5C, Stress responses, Arabidopsis, *Pseudomonas syringae*, Hypersensitive Response, Cell death, Reactive oxygen species (ROS)

## Abstract

**Background:**

Proline (Pro) dehydrogenase (ProDH) potentiates the oxidative burst and cell death of the plant Hypersensitive Response (HR) by mechanisms not yet elucidated. ProDH converts Pro into ∆^1^ pyrroline-5-carboxylate (P5C) and can act together with P5C dehydrogenase (P5CDH) to produce Glu, or with P5C reductase (P5CR) to regenerate Pro and thus stimulate the Pro/P5C cycle. To better understand the effects of ProDH in HR, we studied the enzyme at three stages of the defense response differing in their ROS and cell death levels. In addition, we tested if ProDH requires P5CDH to potentiate HR.

**Results:**

Control and infected leaves of wild type and *p5cdh* plants were used to monitor ProDH activity, *in vivo* Pro catabolism, amino acid content, and gene expression. Wild type plants activated ProDH at all HR stages. They did not consume Pro during maximal ROS accumulation, and maintained almost basal P5C levels at all conditions. *p5cdh* mutants activated ProDH as wild type plants. They achieved maximum oxidative burst and cell death levels producing normal HR lesions, but evidenced premature defense activation.

**Conclusion:**

ProDH activation has different effects on HR. Before the oxidative burst it leads to Pro consumption involving the action of P5CDH. During the oxidative burst, ProDH becomes functionally uncoupled to P5CDH and apparently works with P5CR. The absence of P5CDH does not reduce ROS, cell death, or pathogen resistance, indicating this enzyme is not accompanying ProDH in the potentiation of these defense responses. In contrast, *p5cdh* infected plants displayed increased ROS burst and earlier initiation of HR cell death. In turn, our results suggest that ProDH may sustain HR by participating in the Pro/P5C cycle, whose action on HR must be formally evaluated in a future.

## Background

Proline (Pro) metabolism is sensitive to environmental cues and undergoes particular alterations that contribute to stress tolerance. Plants exposed to dehydration increase Pro synthesis and reduce its catabolism, accumulating high levels of the amino acid. Pro increase has beneficial effects on plant cells and acts as energy reserve to be used upon stress release. Pro metabolism can also affect the cellular redox homeostasis and mobilization of nutrients, which also helps in stress alleviation (for review see 1–3).

Plants synthesize Pro from ornithine and glutamic acid (Glu), involving mostly the second pathway under stress conditions. This process takes place in the cytosol and plastids, involving the action of ∆^1^ pyrroline-5-carboxylate (P5C) synthase (P5CS) and P5C reductase (P5CR) [1,2]. The catabolic route occurs at the mitochondria, where Pro is oxidized into Glu by two enzymes. First, Pro dehydrogenase (ProDH) transforms Pro into P5C. Then, P5C is nonenzymatically converted into glutamate semi-aldehyde that acts as substrate of P5C dehydrogenase (P5CDH) for generation of Glu [1,2]. Oxidation of Pro into Glu is a well-conserved process mediated by enzymes that adopt different organizations in nature. Gram-negative bacteria combine ProDH and P5CDH activities into a single polypeptide, whereas eukaryotes and Gram-possitive bacteria contain monofunctional enzymes [3,4].

ProDH catalyzes the rate limiting step in the oxidation pathway, transferring two electrons from Pro to the non-covalently bound FAD cofactor to subsequently deliver these electrons to a final acceptor [3]. The human ProDH has been early recognized as a tumor suppressor protein regulated by p53, which triggers intrinsic and extrinsic apopototic pathways [5]. The bacterial ProDHs have been well characterized at the kinetic and structural levels by crystalographic studies [3,4]. Unlike these enzymes, the plant ProDH has been mostly studied at the gene expression level, with little functional and biochemical information and no crystal structure being currently available [1,2]. This enzyme resides at the inner mitochondrial membrane where it sustains oxidative phosphorylation and ATP generation [6] using endogenous electron acceptors that still deserve characterization.

Several studies described the sensitivity of Pro metabolism genes to pathogen infections. In flax, *P5CDH* is induced by virulent rust-fungi races that overcome host defense barriers [7]. In Arabidopsis, *P5CS2*, *P5CR* and *ProDH*, but not *P5CDH*, are activated by *Pseudomonas syringae* pv. *tomato* (*Pst*) strains eliciting Effector Triggered Immunity (ETI) and Hypersensitive Response (HR) [8,9]. Under the latter condition, salicylic acid mediates *P5CS2* activation leading to Pro increase at late stages of infection [8]. In *Nicotiana benthamiana*, *P5CS2, P5CR, ProDH* and ornithine δ-amino transferase (*OAT*) genes*,* but not *P5CDH*, are induced by *P. syringae* pv. *tomato* T1 strain eliciting HR-like lesions [10]. Interestingly, *ProDH* and *OAT* were selected by a VIGS-based forward genetic screen by looking for genes regulating non-host resistance [10], and the participation of these enzymes in disease resistance was inferred from the reduction of HR markers in infected tissues of *ProDH-* or *OAT*- silenced plants [9,10].

In animals, yeast, and bacteria, the ProDH activity was associated with the increase in reactive oxygen species (ROS), and was suspected to generate either cellular damage [11] or protective functions [9,12], apparently depending on the ROS levels. Whereas ProDH potentiates ROS and cell death in plant HR-developing tissues [9,10], it extends life span by ROS-mediated activation of antioxidant enzymes in *Caenorhabditis elegans*[12]. It is unclear how ProDH modulates ROS levels and is coordinated with other Pro metabolizing enzymes under these conditions. Its joint action with P5CDH provides energy by charging electrons into the mETC for ATP synthesis, and produces Glu that enters the tricarboxylic acid cycle via α-ketoglutarate [2,3]. ProDH may also operate without P5CDH. This is feasible in yeast, plants and animals containing monofunctional enzymes. In this case, incomplete Pro oxidation can led to P5C increase. Several studies suggested that P5C accumulation can result harmful, apparently by enhancement of ROS levels [13-15]. Interestingly, Arabidopsis plants were found sensitive to exogenous P5C, particularly in the absence of P5CDH where it produced HR-like lesions [14]. However, P5C was also informed as innocuous for this plant [16], raising doubts about the extent and base of its toxicity. More recently, the compound was proposed as an inhibitor of mitochondrial respiration generating lethal superoxide anion levels in yeasts [15].

Functional uncoupling of ProDH and P5CDH not always leads to P5C increase [16]. Alternatively, the P5C produced by ProDH can be used by P5CR to regenerate Pro. Moreover, the joint action of ProDH and P5CR can stimulate the Pro/P5C cycle [5]. As part of this cycle, the human ProDH increases mitochondrial ROS and induces apoptosis, with these effects being abolished by over-expression of mitochondrial MnSOD [5,17]. At present, several features of this cycle (translocation of intermediates, final acceptors of electrons in the mETC, mechanism for mitochondrial ROS generation) remain poorly characterized in plants. However, this circuit seems to operate in the Arabidopsis *p5cdh* mutant, and the *ProDH*- [16] and *P5CS1*- [18] over-expressing plants. As part of this cycle, ProDH may not only promote mitochondrial ROS generation, but also shuttle reducing power from cytosol to mitochondria producing additional redox changes [2,3,5,19]. Finally, ProDH as a flavoenzyme, can eventually transfer electrons to O_2_ as observed for the monofunctional protein of *Thermus thermophilus in vitro*[20].

As described above, many effects could derive from ProDH activation in HR. To start characterizing them, we here analyzed the metabolic context accompanying ProDH stimulation at three different HR stages. We induced HR by inoculating *Pst-AvrRpm1* in Arabidopsis leaves and isolated samples before, during and after the oxidative burst for assessing Pro catabolism, ProDH activity and amino acid content. The studies were performed in wild type plants and the *p5cdh* mutant, used to evaluate how P5CDH affects the ProDH action in HR.

## Results

### Selection of three HR stages for evaluation of ProDH action

**Figure 1:**
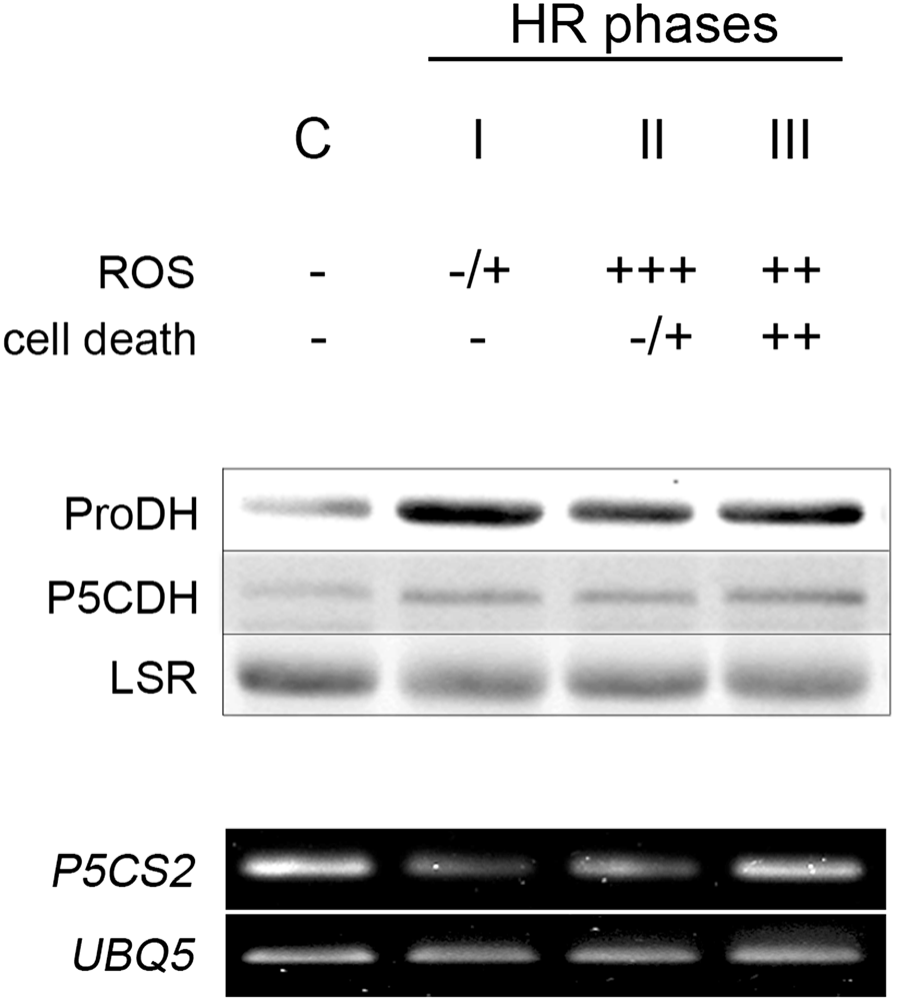
**Pro metabolic enzymes at three phases of HR triggered by *Pst-AvrRpm1*.** Top: Three phases of the HR (I, II, III) having different ROS and cell death levels were selected as indicated in Additional file 1A. C: uninoculated (control) samples. Middle: ProDH and P5CDH content was quantified by Western blots using 30 μg of proteins per sample. Bottom: *P5CS2* gene expression was analyzed by semi-quantitative, and quantitative RT-PCR (Additional file 1B) using primers and conditions described in Additional file 5.

We evaluated ProDH action at different HR stages. In particular, before and during the maximum ROS accumulation that precedes cell death, and at a late HR phase already manifesting cell death. To select these stages, we used conditions that slow HR development, such as infiltration of a moderate dose of bacteria (1–5 × 10^6^ cfu/mL of *Pst-AvrRpm1*) into adult leaves of short-day grown plants. Leaves were excised at 4, 6, 8, 10, 12, 24 hours post-inoculation (hpi) with pathogen or mock solution (MgCl_2_ 10 mM), and monitored for ROS and cell death, as described in Additional file 1A. Mock inoculations generated a transient and mild increase in ROS before 6 hpi and did not affect cell viability at any analyzed stage (not shown). Pathogen treatment began to increase ROS at 6 hpi to produce maximum ROS accumulation at 10 hpi, and trigger cell death since 24 hpi (Additional file 1A; Figure 1). Based on these responses, we selected 6, 10, and 24 hpi for further studies, defining these stages as phases I, II and III of HR.

### Pro metabolic enzymes in HR developing tissues

Total protein extracts from untreated and *Pst*-*AvrRpm1-*treated leaves were analyzed by Western blot assays with anti-ProDH [9] and anti-P5CDH polyclonal antibodies (Additional file 2). A net ProDH increase was evidenced at phase I, and maintained until phase III (Figure 1). Unlike ProDH, P5CDH only showed slight increases during HR. These protein levels were consistent with the abundance of *ProDH* and *P5CDH* transcripts observed under the same conditions (see below). Since *Pst-AvrRpm1* is able to induce the *P5CS2* gene expression at advanced HR stages (since 24 hpi, long-day grown plants; Ref. 8), we monitored its expression in our samples. As observed in Figure 1 and Additional file 1B, we did not detect *P5CS2* transcript accumulation early in HR (up to 24 hpi, short-day grown plants). In turn, expression of *P5CR* and *ProDH2* were found enhanced in HR tissues (Additional file 1C) as reported previously [9].

### Pro catabolism in HR in vivo

**Figure 2:**
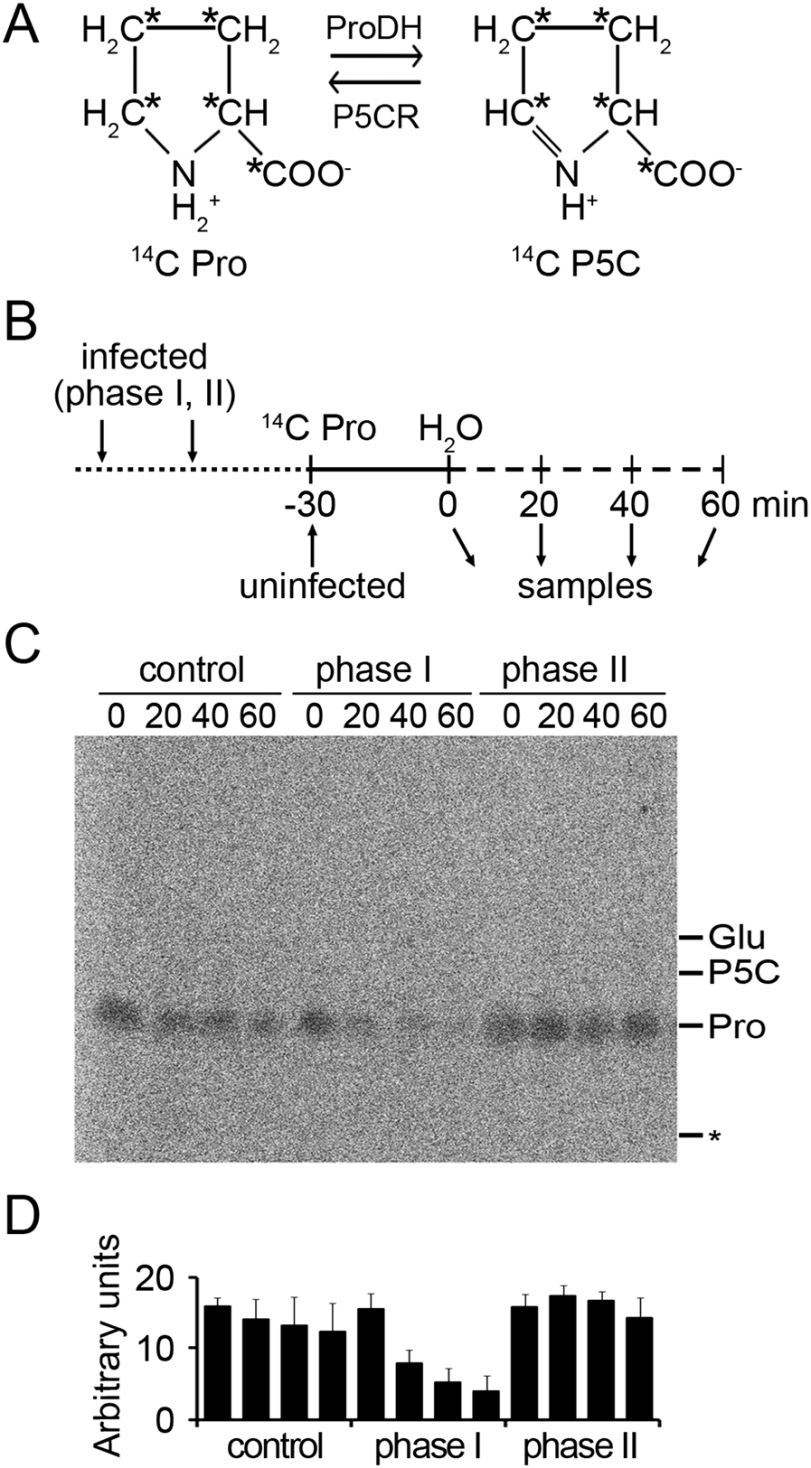
***In vivo ***^**14**^**C-Pro consumption at phases I and II of HR. A**: The ^14^C-Pro molecule used in the assay conserved all labeled carbons (*) after its conversion into ^14^C-P5C, and vice versa. **B**: Scheme of the experimental design applied to evaluate *in vivo*^14^C-Pro consumption in healthy and *Pst-AvrRpm1*-treated leaves (see Methods for details). **C**: PhosphorImager scan of a TLC plate including samples described in B. One representative plate from three independent biological experiments is shown. * indicates the origin of TLC plate. Pro, P5C and Glu positions, determined by ninhydrin staining, are indicated on the right. **D**: Densitometric analysis of the ^14^C-Pro content in the TLC plate using ImageJ software. Data are means ± SD of three independent experiments.

We investigated how ProDH impacts on Pro catabolism before and during ROS accumulation. To this purpose, we fed plants with ^14^C-Pro and monitored its consumption *in vivo*. A Pro molecule labelled at all C residues was used to improve detection of the Pro derivatives (Figure 2A). Uninfected (control) and bacterial-infected leaves excised at phases I and II of HR, were fed with ^14^C-Pro (30 min), transferred to water for different time periods (up to 60 min), and sampled as outlined in Figure 2B. Total amino acids were extracted from these samples and analyzed by TLC (Figure 2C). Consumption of ^14^C-Pro during the water incubation period was evaluated by quantifying the labelled amino acid in this set of samples (Figure 2D).

Healthy tissues had little capacity to catabolize exogenous Pro, as they conserved 83% and 77% of the initial ^14^C-Pro after 40 and 60 min of incubation in water, respectively (Figure 2D). In contrast, tissues at HR phase I showed a proficient ^14^C-Pro consumption, since they preserved 34% and 26% of the amino acid at 40 or 60 min of incubation, respectively (Figure 2D). This increase in Pro catabolism paralleled ProDH accumulation (Figure 1), suggesting it may result from ProDH activation. At phase I, reduction of ^14^C-Pro was not accompanied by appearance of ^14^C-P5C, ^14^C-Glu or other derivatives. Given that the mass of these compounds is directly proportional to their labelling, the ^14^C-P5C and ^14^C-Glu levels would be substantially lower than those of ^14^C-Pro. Therefore, the results suggested that at phase I, ProDH acted with P5CDH to generate ^14^C-Glu which was rapidly metabolized. This possibility was further supported by quantification of amino acids (see below).

Surprisingly, the *in vivo*^14^C-Pro consumption was not maintained at HR phase II (Figure 2C). At this stage, 90% of the amino acid persisted after 60 min incubation (Figure 2D). ProDH activity remained enhanced (Table 1), but had little effect on the Pro levels. Therefore, during maximun oxidative burst P5CDH did not seem to accompany ProDH, suggesting ProDH could act with P5CR under this condition.

**Table 1:** ***In vitro *** **ProDH activity at different HR phases**

	**wild type**		***p5cdh***	
	**- T4C**	**+ T4C**	**- T4C**	**+T4C**
**Control**	0.013 ± 0.001	nd	0.012 ± 0.002	Nd
**Phase I**	0.25 ± 0.09*	0.012 ± 0.001	0.190 ± 0.009*	0.065 ± 0.005
**Phase II**	0.29 ± 0.08*	0.066 ± 0.009	0.240 ± 0.006*	0.038 ± 0.009
**Phase III**	0.34 ± 0.05*	0.053 ± 0.006	nd	Nd

ProDH activity was analyzed on protein extracts of uninoculated (control) and *Pst-AvrRpm1*-inoculated leaves of wild type and *p5cdh* plants. Infected leaves were evaluated at 6, 10 and 24 hours post-infection (phases I, II and III, respectively). Enzyme activity was expressed in nmol of Pro min^-1^ mg of total protein^-1^. Each value is mean ± SD of at least two independent experiments with 20 independent plants.

T4C: L -thiazolidine-4-carboxylic acid, inhibitor of ProDH.

*significant differences respect to uninfected condition (control) of the same genotype (p < 0.05, by *t* test).

### In vitro ProDH activity

We tested if the lack of ^14^C-Pro consumption at phase II resulted from a reduction in ProDH activity. We quantified this activity by using an *in vitro* assay that prevents native conditions favoring functional coupling of ProDH with other enzymes. To this end, the assay previously used to analyze cultured cell extracts [9,21] was adapted to evaluate photosynthetic tissue samples. The new system included two modifications. First, the ProDH activity was determined by monitoring ^3^H-Pro consumption instead of NAD^+^ reduction. The Pro radiolabelled molecule that we selected as substrate contained high specific activity (Additional file 3 and Methods). Second, the reaction was developed at neutral instead of alkaline pH, to avoid the reverse P5CR activity [22,23], preserving the ProDH activity [6]. The new assay, conserved the use of L-thiazolidine-4-carboxylic acid (T4C) as competitive ProDH inhibitor, and resulted suitable to determine ProDH activity in Arabidopsis leaf extracts (Table 1)*.*

As expected, wild type plants increased the ProDH activity at HR phase I (19-fold respect to basal conditions; Table 1). Clearly, the enhancement of enzyme activity was preserved at phase II, and slightly increased at phase III. Therefore, the results suggested that *in vivo* ProDH remained active at all three HR phases.

### Changes in amino acid levels

To further evaluate the coordinated action of ProDH with P5CDH or P5CR, we quantified the content of Pro and its derivatives at each HR stage. Alterations in levels of these compounds were indicative of the plant enzyme activities under stress [16]. We included in this study the *A. thaliana* T-DNA insertion line Salk_021026 previously described as a *p5cdh* knock-out mutant plant [24]. As expected, this plant neither accumulated P5CDH protein nor *P5CDH* transcripts in leaf or floral tissues (Additional file 2). Interestingly, the *p5cdh* infected tissues retained the capacity to activate ProDH in response to *Pst*-*AvrRpm1* reaching similar activity levels than infected wild type tissues (Table 1). Therefore, this plant resulted useful to detect responses dependent on P5CDH, but not ProDH, activity.

By HPLC assays we quantified 19 amino acids on naive and infected tissues of wild type and the *p5cdh* plants. At basal conditions, all amino acids had similar levels in both plants (p < 0.05 by *t* test), except Pro which was increased in the mutant (64%; Table 2). After bacterial treatment all amino acids, except Ser, altered their content in at least one HR phase in one genotype, showing significant differences respect to the uninfected condition (p < 0.05 by *t* test) (Table 2). Most of them (Arg, Tyr, Val, Phe, His, Ile, Lys, Leu, Gly, Asn, Thr, Ala) displayed a progressive increase in both plants, which was generally lower in the mutant, suggesting that P5CDH may contribute to accumulation of these amino acids in infected wild type tissues. GABA was included in this group based on its large increase at HR phase II, which was more pronounced in the mutant. In contrast, Glu and Asp were reduced by infection in both plants. In turn, P5C, Pro and Gln exhibited different alterations in both genotypes.

**Table 2:** Amino acid content at different HR phases

	**wild type**				***p5cdh***			
	**C**	**I**	**II**	**III**	**C**	**I**	**II**	**III**
**Ser**	872 ± 129	840 ± 160	948 ± 98	1387 ± 150	895 ± 96	1013 ± 65	891 ± 19	820 ± 70
**Arg**	7.2 ± 0.9	11.2 ± 0.5*	19 ± 4*	62 ± 7*	7.1 ± 0.9	9.1 ± 1.2*	19 ± 4*	34 ± 6*
**Tyr**	10 ± 1	21 ± 1*	34 ± 6*	78 ± 7*	11 ± 1	17 ± 1*	39 ± 3*	33 ± 5*
**Val**	59 ± 8	173 ± 17*	171 ± 14*	440 ± 76*	59 ± 4	102 ± 10*	188 ± 22*	223 ± 44*
**Phe**	28 ± 5	98 ± 6*	108 ± 22*	272 ± 36*	29 ± 3	56 ± 4*	118 ± 12*	127 ± 27*
**His**	13 ± 2	26 ± 5*	37 ± 6*	87 ± 16*	16 ± 3	28.7 ± 1.4*	35 ± 5*	38 ± 4*
**Ile**	17 ± 4	56 ± 10*	58 ± 12*	228 ± 45*	20 ± 2	40 ± 3*	78 ± 7*	50 ± 7*
**Lys**	9 ± 2	20 ± 4*	23 ± 5*	92 ± 19*	10 ± 2	18 ± 4*	38 ± 5*	#
**Leu**	25 ± 3	95 ± 8*	115 ± 24*	408 ± 59*	22 ± 3	51 ± 9*	128 ± 9*	169 ± 36*
**Gly**	230 ± 38	280 ± 48*	368 ± 42*	708 ± 31*	284 ± 17	259 ± 7*	588 ± 32*	531 ± 74*
**Asn**	75 ± 16	124 ± 5*	145 ± 20*	134 ± 24*	90 ± 15	95 ± 18	128 ± 19	158 ± 17*
**Thr**	300 ± 43	497 ± 35*	449 ± 46*	733 ± 74*	366 ± 55	459 ± 32*	464 ± 60*	#
**Ala**	337 ± 46	433 ± 87*	391 ± 45*	618 ± 28*	318 ± 23	410 ± 31*	425 ± 15*	299 ± 57
**GABA**	19 ± 2	30 ± 3*	164 ± 23*	11 ± 1*	13 ± 3	19 ± 4*	291 ± 37*	4 ± 1*
**Glu**	693 ± 60	701 ± 49	812 ± 89	279 ± 49*	742 ± 83	833 ± 77	684 ± 51	523 ± 43*
**Asp**	285 ± 41	228 ± 48	300 ± 24	62 ± 15*	324 ± 8	401 ± 12*	202 ± 26*	114 ± 12*
**P5C**	1.3 ± 0.2	1.4 ± 0.3	1.4 ± 0.3	1.6 ± 0.2	1.2 ± 0.2	1.3 ± 0.1	1.9 ± 0.3*	1.4 ± 0.3
**Pro**	606 ± 106	376 ± 74*	572 ± 109	1393 ± 169*	995 ± 134 ^a^	951 ± 201	941 ± 87	1112 ± 22*
**Gln**	916 ± 100	1114 ± 239*	718 ± 31*	764 ± 92	785 ± 70	838 ± 89*	836 ± 36	437 ± 97*

C:uninfected samples (control); I, II, III: *Pst-AvrRpm1*-infected samples isolated at phases I, II or III of HR.

*Significant differences between infected and control samples of the same genotype (p < 0.05 by *t* test).

#Samples analyzed in different experiments containing similar values similar to those of wild type plants.

^a^Significant differences regarding the wild type control value (p < 0.05 by *t* test).

Values are expressed as nmol/g fresh weight. Each value is mean ± SD of 3 independent experiments (10 leaves per plant, 6 plants per experiment).

**Figure 3:**
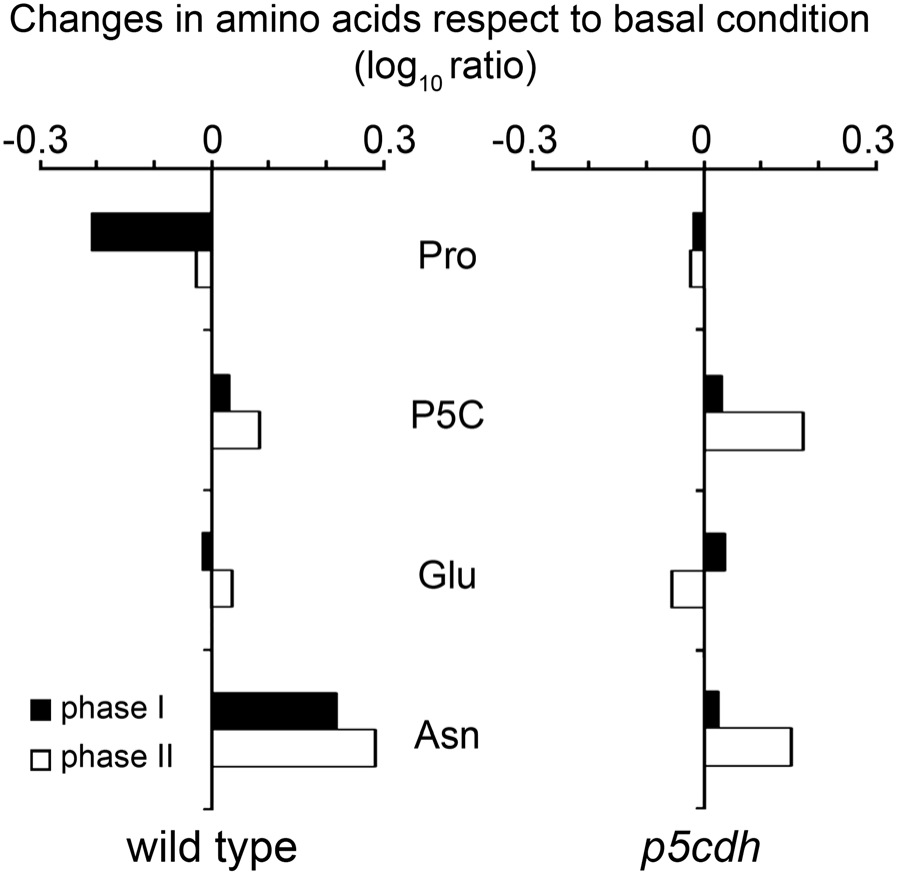
**Changes in the amino acid content in wild type and *p5cdh *plants.** Based on the absolute values shown in Table 2, the ratio between the content of Pro, P5C, Glu or Asn in infection (phases I and II) and basal condition (control) was log transformed (base 10) to compare the responses of wild type and *p5cdh* plants. Statistical analysis of data is described in legend of Table 2.

To analyze in greater depth the behavior of Pro, P5C and Glu at phases I and II of HR, we compared their alterations in wild type and mutant plants (Figure 3). Glu is a central molecule in plant amino acid metabolism contributing to the synthesis of several of these compounds. Its α-amino group can be transferred to oxalic acid to form Asp, a precursor of Asn and the aspartate family of amino acids [25]. Therefore, we included Asn in the analysis, as a terminal amino acid indirectly derived from Glu. At phase I, wild type plants reduced Pro and increased Asn without altering P5C or Glu (Figure 3). Once again, the results suggested that ProDH and P5CDH acted together at phase I, generating Glu and thus contributing to Asn increase (Additional file 4). Consistently with this possibility, Pro consumption was not accompanied by Glu accumulation at phase I (Figure 2). Moreover, *p5cdh* plants were unable to reduce Pro or increase Asn at this stage (Figure 3).

At phase II, wild type tissues recovered their basal Pro content and maintained Asn at similar levels than phase I, while they preserved basal P5C and Glu content. Meanwhile, *p5cdh* plants showed just a modest increase of P5C and Asn (Figure 3; Table 2). As observed in our previous studies, phases I and II exhibited differences in Pro catabolism (Additional file 4), with the responses observed in wild type plants at phase II (arrest in both Pro consumption and Asn increase, and maintenance of P5C) being consistent with stimulation of the Pro/P5C cycle.

Importantly, wild type plants did not accumulate P5C during the initial 24 hpi with *Pst-AvrRpm1* (Figure 3). As described, the oxidative burst and cell death were produced during this period (Figure 1; Additional file 1A).

### ProDH action in the p5cdh mutant

**Figure 4:**
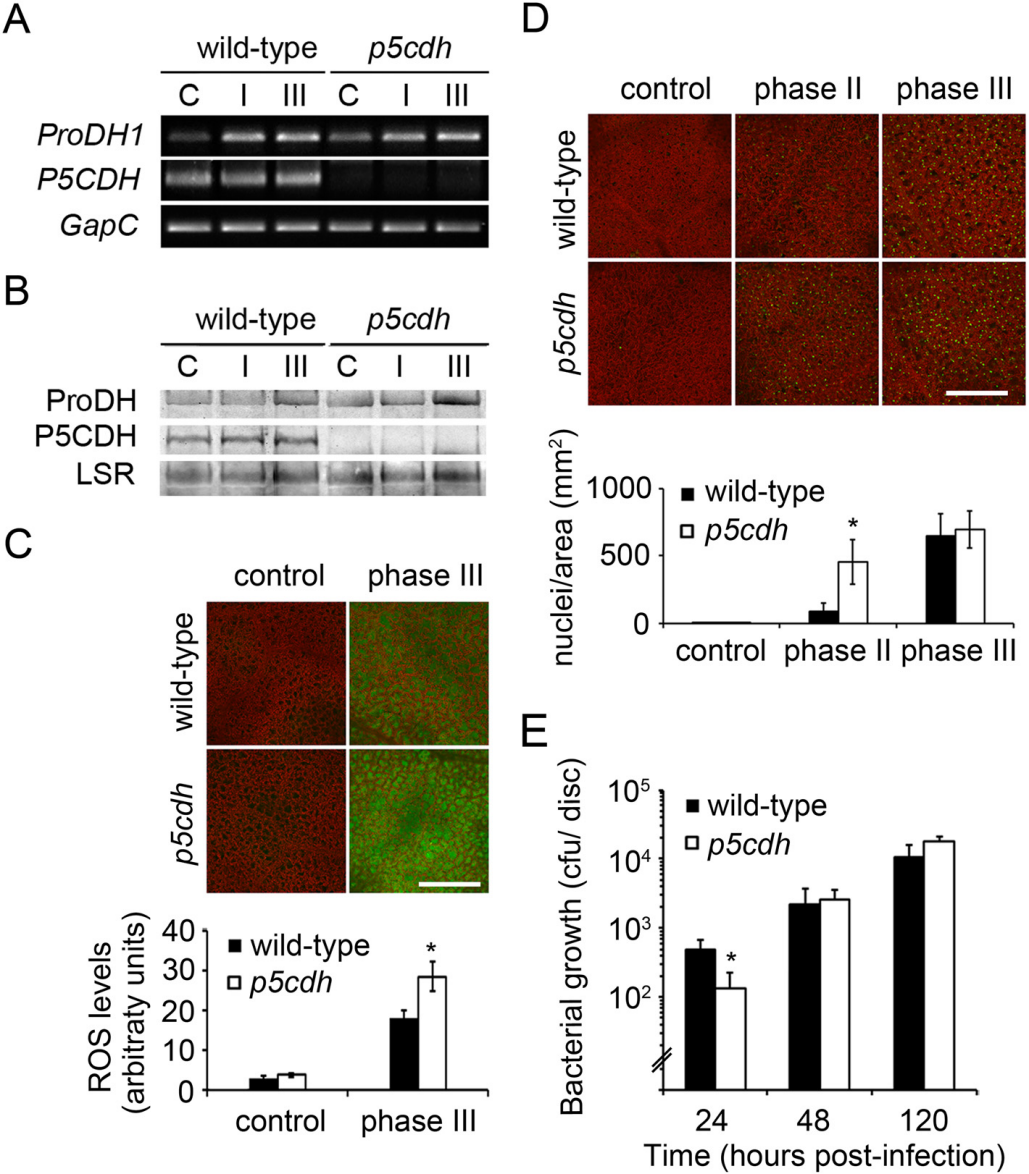
**HR features are manifested early in the *p5cdh *mutant.** Comparison of responses of wild type (wt) and *p5cdh* plants to infection by *Pst-AvrRpm1*. The abundance of ProDH and P5CDH transcripts **(A)** and proteins **(B)** was determined as described in the legend of Figure 1. **C**: ROS levels analyzed by H_2_DCF-DA staining. **D**: Nuclei from non-viable cells detected with SYTOX Green dye. In **C** and **D** probe fluorescence was quantified with ImageJ software (bottom). Values represent mean ± SE of samples (3 infection experiments, 3 plants per genotype each; 32 images per genotype in **C**; and 18 in **D**). **E**: *Pst-AvrRpm1* content *in planta* at the indicated times post-inoculation. Data represent means ± SD of one representative from 3 experiments. Control: uninoculated samples; I, II, III: samples corresponding to phases I, II, III of HR. Bars = 400 μm. *: significant differences between samples from wild type and mutant plants (p < 0.05 by *t* test).

We wondered if ProDH requires P5CDH to potentiate HR. To evaluate this issue, we monitored HR features in *Pst-AvrRpm1-*infected *p5cdh* leaves. These tissues lacked P5CDH protein and transcripts but accumulated ProDH transcripts and protein as infected wild type tissues (Figure 4A,B). As mentioned before, the *p5cdh* plant also preserved the capacity to activate ProDH in response to *Pst-AvrRpm1* (Table 1). Therefore, this mutant was suitable to test the effect of ProDH in HR, in the absence of P5CDH. As in previous studies, we evaluated ROS and cell death as HR markers, and susceptibility to avirulent bacteria as parameter of plant resistance [9]. The increase of ROS was higher in the mutant (Figure 4C), which triggered cell death before wild type plants (Figure 4D). In addition, the *p5cdh* plants showed enhanced pathogen resistance at 24 hpi (Figure 4E). However, at later infection stages these differences were no longer observed (Figure 4D, E). Therefore, the absence of P5CDH affected the timing, but not strength, of HR producing a premature increase of ROS and cell death, and transient enhancement of pathogen resistance.

## Discussion

### ProDH activation in HR

We used an *in vitro* assay to quantify ProDH activity in Arabidopsis leaf tissues. The methods monitoring Pro-dependent NAD^+^ reduction by spectrophotometry were not useful to this end since pigments from photosynthetic tissues interfered with NADH detection [9]. We overcome this limitation by following ^3^H-Pro consumption instead of NAD^+^ reduction. Under basal conditions wild type plants consumed 13.4 cpm ^3^H-Pro min^-1^ mg protein^-1^, equivalent to 33 nmol ^3^H-Pro min^-1^ mg protein^-1^. This value was in agreement to that reported for the enzyme in liver mitochondrial extracts (4.3 nmol min^-1^ mg protein^-1^), where ^3^H-Pro labelled at the C-5 was used as substrate, and transference of ^3^H to water was monitored [26]. The increase of ProDH activity detected in the infected leaves (19–26 times over basal level; Table 1) far exceeded the one observed in extracts of cultured cells challenged with the same pathogen (30% increase over basal levels; Ref. 9). This difference was probably due to greater responsiveness of leaf tissues to pathogen, and improvement of the assay.

Strong and persistent ProDH activation (Table 1) coexisted with transient Pro decrease (Figure 3) in infected tissues, indicating that the greater enzyme capacity did not substantially impact on the Pro levels. Interestingly, these tissues induced ProDH without a previous Pro increase. Something similar was observed for the *p5cs1* plants that even with low Pro levels, retained the ability to activate *ProDH* after stress release [27]. Moreover, under cold acclimation, *ProDH* induction was accompanied by Pro increase [28]. One interesting observation derived from our studies was the maintenance of ProDH activation in the absence of P5CDH (Table 1), which was already noticed on *p5cdh* plants recovering from drought stress [16]. Therefore, ProDH activation occuring after stress release would not always be accompanied by P5CDH and could have minor effects on the Pro content.

Although *Pst-AvrRpm1* induced *ProDH* and *P5CS2* genes at advanced stages of HR (≥24 hpi, plants grown under long-day conditions; Ref. 8), it only activated *ProDH* at the early HR phases (6, 10, 24 hpi, plants grown under short-day conditions; Figure 1). Thus, in contrast with rehydration, the HR condition stimulates Pro catabolism before Pro synthesis. How ProDH remains active without *de novo* Pro synthesis under these circumstances is unknown. Transport of Pro from healthy tissues [29], stimulation of the Pro/P5C cycle [16,18], or both responses can take place there. Restoration of basal Pro (Figure 3) without *P5CS* induction (Figure 1; Additional file 1B) observed at phase II in wild type plants was consistent with the first possibility. As discussed below, several additional results supported the latter one.

### P5C levels in the HR lesion

The functional uncoupling of ProDH and P5CDH may generate P5C accumulation with toxic effects for plants [14]. In yeast, the P5C increase from 200 to 300 μM results harmful for stressed cells [15]. Our previous finding that *Pst-AvrRpm1* activated *ProDH* but not *P5CDH*, suggested that P5C could accumulate and signal HR cell death [8]. In previous studies, we did not detect P5C increase by spectrophotometric assays in wild type tissues developing HR [9]. In the current work, we used HPLC to unequivocally determine the P5C content in HR. In addition, we analyzed *p5cdh* infected tissues that are prone to accumulate P5C due to ProDH activation. We detected a transient increase of P5C at HR phase II in *p5cdh* plants (58% over basal level; Table 2) that paralleled cell death initiation (Figure 4D). How P5C contributes to HR cell death in *p5cdh* plants is unknown. However, it is clear that P5C does not signal ROS generation or cell death initiation in the HR lesion of wild type plants, since its levels remain almost constant at all phases of this response (Table 2).

### Pro catabolism at phases I and II of HR

*In vitro* assays evidenced similar ProDH activation at all HR stages (Table 1). At difference, *in vivo* studies, and amino acid quantification, showed variations in Pro catabolism at phases I and II. At phase I, Pro consumption (Figures 2 and 3) was accompanied by increase of the terminal Glu-derivative Asn (Figure 3), suggesting that basal Pro was transformed into Glu. At phase II, Pro did not continue to decline, and Asn was not further increased (Table 2; Figures 2 and 3). The alteration of Pro catabolism manifested in phase II may have different causes.

One possibility is that enzyme and substrate have different partitioning. This is, however, unlikely since mitochondrial Pro depletion has not been reported to date [1,30]. Alternatively, plant- or bacterial-derived compounds may inhibit ProDH at phase II. Nevertheless, the existence of such products has not been reported. P5C, which may eventually lead to product inhibition, did not increase under this condition in wild type plants (Figure 3). Furthermore, since ProDH preserved its catalytic capacity *in vitro* until phase III (Table 1) irreversible enzyme inhibition can be discarded. Another possibility is that ProDH acts with P5CR recycling Pro and P5C. Recent studies propose that the Pro/P5C cycle operates in Arabidopsis tissues exposed to abiotic stress, and probably contributes to exogenous Pro toxicity [14,16,17]. The results that suggest stimulation of this cycle at HR phase II are: *i*) co-existence of ProDH activation (Table 1), and unaltered Pro and P5C levels (Figure 3); *ii*) absence of *P5CS* induction at the time of *ProDH* activation (Figure 1, Additional file 1B); *iii*) premature accumulation of ROS in *p5cdh* infected tissues able to induce ProDH (Figure 4; Table 1). Moreover, the joint activation of *ProDH* and *P5CR* genes here observed (Figure 1; Additional file 1C) was consistent with previous results [9] and also supported this possibility. Therefore, although the Pro/P5C cycle requires formal demonstration in plants, the current knowledge about Pro metabolism [1-3,16,18,19] and our results are consistent with stimulation of this cycle in HR.

### ProDH action at HR Phase II

Arabidopsis transgenic plants that reduced, but not eliminated, ProDH expression attenuated the oxidative burst, hypersensitive cell death, and resistance to *Pst-AvrRpm1*[9]. In contrast, the null *p5cdh* mutant did not reduce these responses (Figure 4). This suggests that ProDH does not require P5CDH to potentiate those HR features. Consistently, ProDH had no effect on Pro consumption at the time of maximum ROS accumulation (phase II; Figure 3). Moreover, P5CDH may even prevent or reduce the capacity of ProDH to produce ROS and associated defenses. In this sense, infected *p5cdh* plants prematurely stimulated ROS burst and cell death (Figure 4). Moreover, induction of *P5CDH* was observed in diseased, but not resistant, cultivars of flax [7], nor in Arabidopsis or *N. benthamiana* plants triggering ETI or HR non-host resistance [8-10]. Curiously, P5CDH was not required for salt stress tolerance in Arabidopsis [14], suggesting this enzyme would neither accompany ProDH under such condition.

The participation of ProDH in the Pro/P5C cycle may have several implications for HR. The cycle shuttles reducing power into mitochondria, coupling cytosolic NADPH oxidation with mETC activity. Under an excess of electrons, it may potentiate mitochondrial ROS accumulation, as occurring in human cells over-expressing ProDH [5]. The cycle may also alter the cytosolic NADPH/NADP^+^ ratio and therefore affect other redox buffers, such as glutathione and ascorbate, intimately associated to biotic defenses [31-35]. Indeed, cytosolic redox alterations in concert with plasma membrane NADPH oxidase define the output of pathogen resistance responses [36]. Therefore, as part of the Pro/P5C cycle, ProDH may potentiate plant immunity by supplying energy or altering the mitochondrial or cytosolic redox homeostasis.

### Changes in the metabolism of other amino acids may also affect HR development

Several studies evaluated the amino acid changes occurring in Arabidopsis leaf tissues infected with virulent *Pst*, virulent *Pseudomonas syringae* pv *maculicola* ES4326 (*Psm*) and other pathogens [37-40], but none of them described the effects of *Pst-AvrRpm1* which were reported in the current work.

Reduction of Glu and increase of Gly, Thr, Arg, Val, Ile, Lys, Leu and Phe, His, and GABA (Table 2), are triggered by virulent *Pst* DC3000, the TTSS-deficient strain *Pst* DC3000 hrpA [39], and *Pst*-*AvrRpm1* (Table 2). Similar changes, excepting those affecting Glu, Thr and Arg, are induced by *Psm*[40], suggesting their association with plant basal defenses. In contrast, Tyr accumulation was stimulated by *Pst-AvrRpm1* (Table 2), *Pst* DC3000 [39], *Psm*[40], but not by *Pst* DC3000 hrpA [39], probably involving ETI. The Lys and Asp alterations here described (Table 2) are similar to those accompanying the accumulation of pipecolic acid in *Psm*-infected tissues [40]. This Lys derivative functions as inducer of systemic acquired resistance, basal resistance, and resistance to *Psm-AvrRpm1*. Interestingly, pathways affecting the metabolism of Pro, Lys, Asp, and pipecolic acid have been associated with regulation of plant immunity and pipecolic acid (homoproline) and Pro are proposed to involve similar biosynthetic pathways [41].

One of the most notorious changes involved GABA. This Glu derivative, as Pro, is a compatible osmolyte that accumulates under different stress conditions, including microbial infections [42]. GABA is a source of N and C for the pathogen, and an inducer of plant antioxidant enzymes [39,43]. Conversely, accumulation of the GABA derivatives succinic semialdehyde and γ-hydroxybutyrate has been associated to ROS increase [44]. In the interaction of Arabidopsis with *Pst* DC3000, GABA represses the expression of pathogen effectors weakening pathogen virulence [39,43]. In response to *Pst-AvrRpm1*, a strong and transient GABA increase paralleled the oxidative burst (phase II). Such increase was more pronounced in the *p5cdh* mutant (Table 2) where GABA may help to counteract bacteria with little or no effect on anti-oxidant responses, since this plant reduced pathogen propagation displaying enhanced ROS (Figure 4). Interestingly, in infections by *Agrobacterium tumefaciens*, Pro antagonizes the GABA-mediated effects on quorum-sensing and pathogen virulence [45], whereas their coordinated effects on other plant-pathogen interactions remain to be studied.

## Conclusions

This is the first study describing the effects derived from ProDH activation in plant tissues generating HR. We report that ProDH remains activated during HR progression in Arabidopsis leaves treated with *Pst-AvrRpm1*, to generate different Pro metabolic changes before and after ROS burst. In the first case, ProDH contributes to Pro consumption by acting with P5CDH (Additional file 4). During maximal ROS accumulation, ProDH has minor effect on the Pro, P5C, Glu or Asn levels, indicating its functional uncoupling to P5CDH. Interestingly, the *p5cdh* null mutant retains the capacity to activate ProDH in HR, offering a suitable model to study functional uncoupling of ProDH and P5CDH in plants. P5CDH was found dispensable for generation of HR cell death and oxidative burst, but required for normal timing of defense activation. In addition, P5C accumulation was not involved in HR cell death signaling, at least in wild type plants. Therefore, based on the current knowledge about plant Pro metabolic enzymes, our results suggest that the main effect of ProDH in HR can derive from its joint action with P5CR.

## Methods

### Plant growth and bacterial treatment

*Arabidopsis thaliana* L. Heynh ecotype Col-0 plants were grown in soil under a 10 h light/14 h dark period at 22°C and used at the age of 6 weeks. The *p5cdh* mutant line Salk_021026 was obtained from ABRC. Segregation analysis of Km resistance revealed a single T-DNA insertion, whose position at the P5CDH loci was verified by PCR using the SIGnAL T-DNA express iSecTool (Salk Institute Genomic Analysis Laboratory) recommended primers. *P. syringae* pv. *tomato* DC3000 *AvrRpm1* (*Pst-AvrRpm1*) was grown and inoculated on expanded leaves for activation of HR (1–5 × 10^6^ cfu/mL) or bacterial growth analysis (5 × 10^5^ cfu/mL), as reported previously [9].

### Gene expression

RNA was extracted and used on semi-quantitative [8,9] or quantitative [46] RT-PCR with primers and conditions described on previous works and Additional file 5.

### Protein quantification

P5CDH rabbit polyclonal antibodies were raised against the synthetic 23-amino acid N-terminal peptide FATVDAEELSGAHPAEVQSFVQG (Sigma Co). Goat anti-rabbit IgG IRDye 800CW (LI-COR Bioscience) was used as secondary antibody. Membranes were scanned with Odyssey Infrared Imaging System (LI-COR Biosciences) on channel 800 for secondary antibody. Detection of large subunit of RuBisCo (channel 700) was monitored to control loading. Generation of ProDH antibodies and protein extraction are described elsewhere [9].

### Amino acid quantification

After extraction of leaf tissues with 80% v/v ethanol at 80°C for 60 min, supernatants were evaporated to dryness and resuspended in miliQ water. Amino acids were derivatized with the fluorescent reagent 6-aminoquinolil-N-hidroxisuccinimidilcarbamato (ACQ; prepared at IPK, Gatersleben, Germany), run on reverse phase HPLC (2795 Alliance, Waters GmbH, Germany), and analyzed as described previously [47].

### ^14^C feeding experiments

Uninfected and *Pst-AvrRpm1-*infected leaves were incubated in 40 μL of water containing 1 nCi ^14^C-Pro (L-^14^C (U) Proline 266 mCi/mmol; #NEC258E, Perkin Elmer) for 30 min at 22°C before being washed and transferred to water for different time periods (0, 20, 40, 60 min), and then dried on tissue paper, weighed, and frozen in liquid N_2_. Amino acids were extracted with methanol:chloroform:water (12:5:3 v/v) according to Boggess and col. [48]. Extracts were dried and resuspended in miliQ water (50 μL). Aliquots (5 μL) of samples were separated by TLC using silica gel 60 (Merck, Germany) and 80% v/v ethanol as the mobile phase. These conditions allowed separate Pro, P5C and Glu. Plates were dried and analyzed on PhosphorImager (Fujifilm FLA3000, Amersham Biosciences). Unlabelled Pro, Glu (SIGMA) and P5C (kindly provided by Dr. JM Phang; National Cancer Institute, Frederick, Maryland, USA), were used as standards. Ninhydrin staining [49] was used to control equal loading.

### Enzymatic assays

Leaves were frozen and pulverized under liquid nitrogen to be treated at 4°C with extraction buffer (100 μl/ 50 mg tissue; 50 mM Tris–HCl pH = 7.4, 7 mM MgCl_2_, 600 mM KCl, 3 mM EDTA, 1 mM DTT, 1 mM PMSF, 5% [w/v] PVP), and then centrifuged at 13000 *g* for 10 min at 4°C. The supernatant was used to determine protein content (Bradford assay) and ProDH (EC 1.5.99.8) activity was determined using a modified version of the method described by Kant and col. [21]. Briefly, 10–100 μL of protein extracts were incubated in 100 μL of reaction mixture containing 50 mM Tris–HCl pH = 7.4, 1 mM NAD^+^, 25 mM Pro, 1 nCi ^3^H-Pro [L-(2,3,4,5- ^3^H) Proline 94.4 Ci/mmol; #NET483V, Perkin Elmer; Additional file 3] at 25°C for up to 120 min. In parallel one aliquot of each sample was treated with the ProDH competitive inhibitor L-thiazolidine-4-carboxylic acid (T4C; 1 mM) [50]. The reaction was stopped by adding 1 vol of methanol:chloroform:water mix (12:5:3) and after evaporation, samples were analyzed by TLC as describe above. Bands corresponding to ^3^H-Pro were scraped and mixed with scintillation cocktail (OptiPhaseHighsafe 3; Perkin Elmer) to quantify radioactivity in a scintillation counter (1214 Rackbeta, LKB Wallac, Pharmacia, Turku, Finland).

## Abbreviations

P5C: ∆^1^ pyrroline-5-carboxylate; P5CS: ∆^1^ pyrroline-5-carboxylate synthase; P5CR: ∆^1^ pyrroline-5-carboxylate reductase; H2 DCF-DA: dichlorodihydrofluorescein-diacetate; ETI: Effector triggered immunity; Glu: Glutamic acid; hpi: Hours post-inoculation; HR: Hypersensitive response; mETC: Mitochondrial electron chain; OAT: Ornithine δ-aminotransferase; Pro: Proline; ProDH: Proline dehydrogenase; Pst-AvrRpm1: *Pseudomonas syringae* pv. *tomato AvrRpm1*; ROS: Reactive oxygen species; T4C: L-thiazolidine-4-carboxylic acid.

## Competing interests

The authors declare that they have no competing interests.

## Authors’ contributions

MIM, YSR and NMC analyzed gene expression, proteins and amino acids content, ProDH activity, ROS and cell death levels and pathogen proliferation. MEA and M-RH designed and supervised the study. All authors discussed the results, prepared and approved the final version of the manuscript.

## Authors’ information

MIM and YSR are CONICET fellows. MEA is a senior Career Investigator of CONICET.

## Supplementary Material

Additional file 1 **Three phases of HR selected to study Pro metabolism genes. A**: Cell death (top) and ROS (bottom) levels at phases I, II and III of HR determined by SYTOX Green [9] and diamino benzidine staining [46], respectively. **B**: *P5CS2* expression analyzed by qRT-PCR according to Fabro et al. [46]. Values were obtained applying the ΔΔCt method. Bars represent average ± SD from three replicates. **C**: *P5CR* and *ProDH2* expression analyzed by sqRT-PCR. Primers and conditions used in B and C are described in Additional file 5. *UBQ5* was used as internal control. Click here for file

Additional file 2 **Validation of anti-P5CDH antibodies.** Polyclonal rabbit anti-P5CDH antibodies were used on Western blot assays to analyze total protein extracts from flowers or leaves of wild type (wt) or *p5cdh* mutant plants. Samples were loaded on 10% SDS-PAGE gels and analyzed with anti-P5CDH (1/300) and secondary goat anti-rabbit (1/20000) antibodies. Membranes were scanned with Odyssey Infrared Imaging System (LI-COR Biosciences) for detection of secondary antibody (green) and RuBisCo (red). Merge of both channels is shown at the bottom. LSR: large subunit of RuBisCo. Click here for file

Additional file 3 **Radiolabelled **^**3**^**H-Pro molecule used as substrate for quantification of ProDH activity *in vitro, *** **and its transformation into P5C.** Click here for file

Additional file 4 **Presumable action of ProDH in phases I and II of HR.** The results of this study suggest that ProDH acts in two different metabolic contexts throughout HR. Prior to oxidative stress (phase I) the enzyme likely acts together with P5CDH producing complete Pro oxidation. These enzymes may somehow become uncoupled at the stage of oxidative stress (phase II), where ProDH remains active but does not contribute to Pro consumption or P5C accumulation. Transcriptional activation of P5CR suggests a coupling of ProDH and P5CR at this second phase with consequent stimulation of the Pro/P5C cycle. Click here for file

Additional file 5 **Primers and conditions used for sqRT-PCR experiments.** # Melting temperature used for each pair of primers. *For each pair of primers the optimal cycle number was selected from a linear amplification range using the following conditions: 1 μg RNA, 500 ŋg random hexamer primers, 200 U M-MLV reverse transcriptase for cDNA synthesis; 1 μl cDNA, 10 μM primer, 25 μl final volume for PCR. The *GapC* gene, insensitive to *Pst* treatment (Arabidopsis eFP Browser), was used as internal control. Click here for file
