# Supplementary figures and images for: Context of action of Proline Dehydrogenase (ProDH) in the Hypersensitive Response of Arabidopsis
Source: BMC Plant Biol. 2014 Jan 13;14:21. doi: 10.1186/1471-2229-14-21 (PMC3902764; doi:10.1186/1471-2229-14-21)

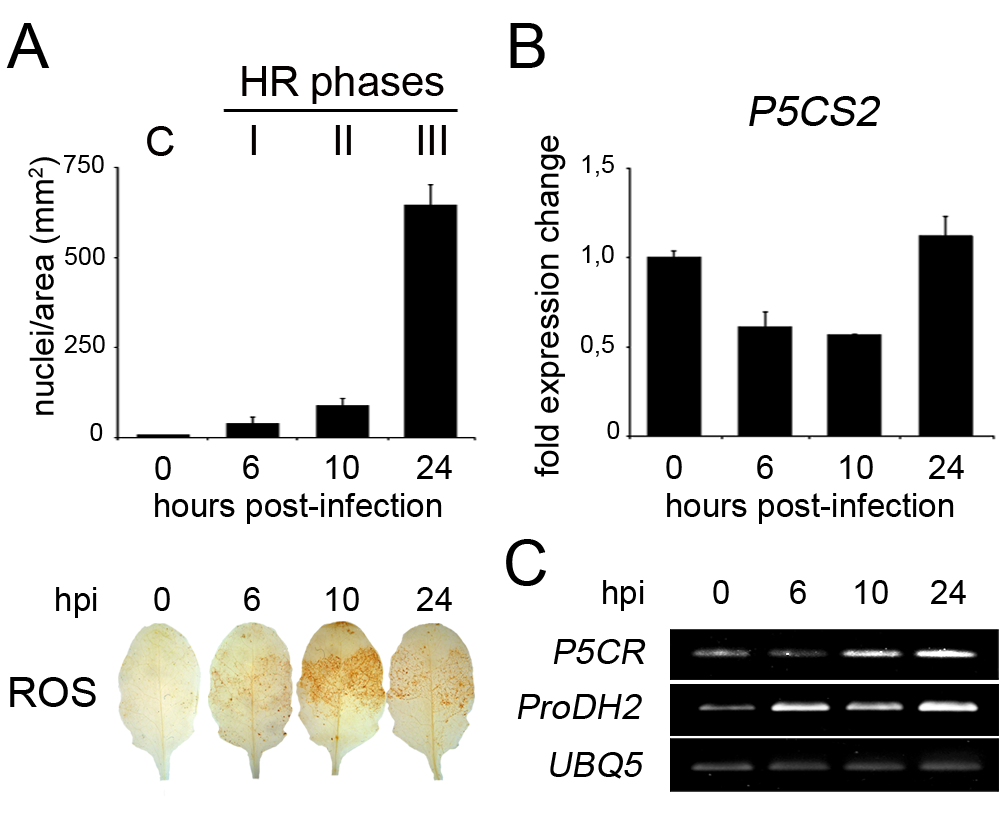

Supplement: Additional file 1 — Three phases of HR selected to study Pro metabolism genes. A: Cell death (top) and ROS (bottom) levels at phases I, II and III of HR determined by SYTOX Green [9] and diamino benzidine staining [46], respectively. B: P5CS2 expression analyzed by qRT-PCR according to Fabro et al. [46]. Values were obtained applying the ΔΔCt method. Bars represent average ± SD from three replicates. C: P5CR and ProDH2 expression analyzed by sqRT-PCR. Primers and conditions used in B and C are described in Additional file 5. UBQ5 was used as internal control. [file 1471-2229-14-21-S1.tiff]

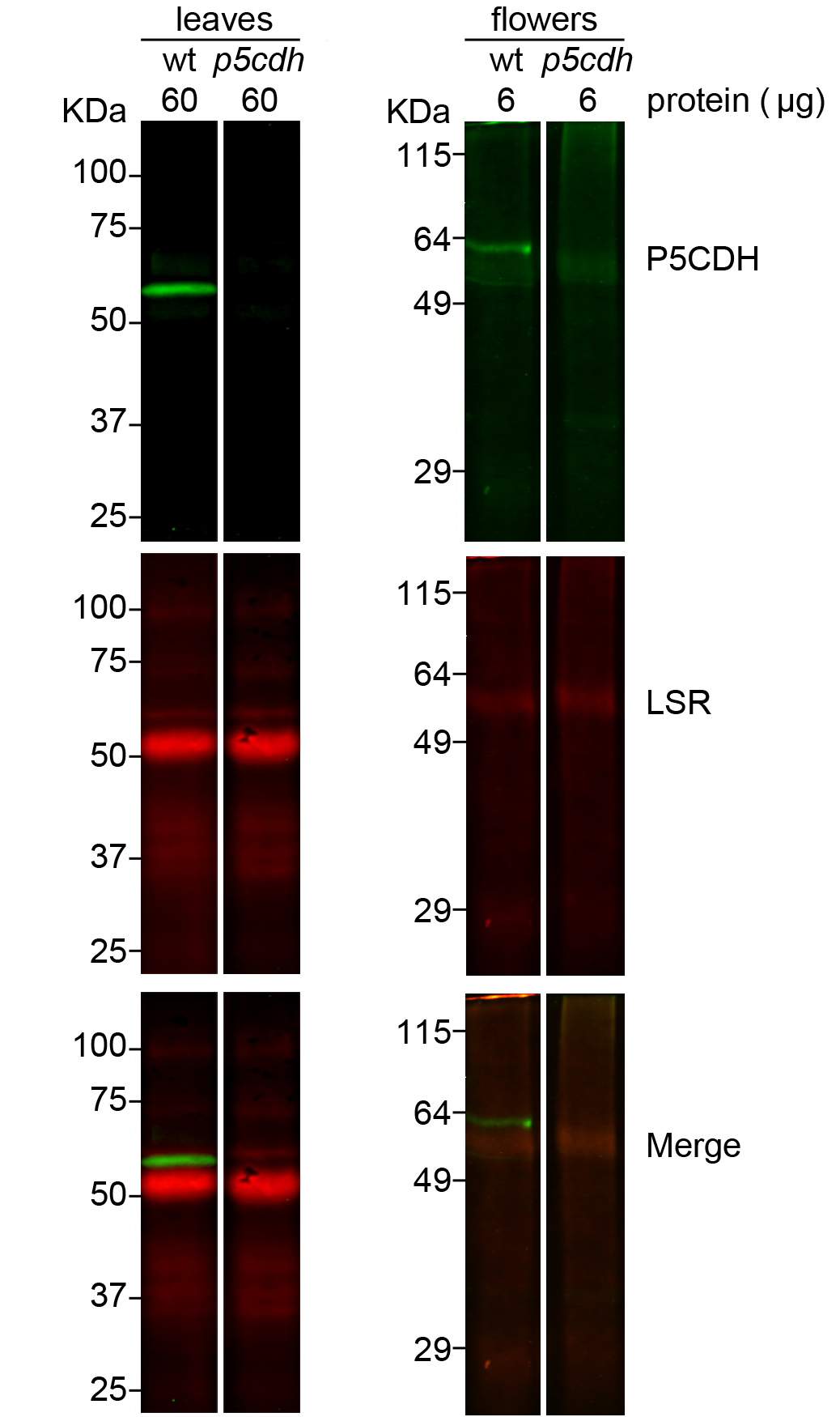

Supplement: Additional file 2 — Validation of anti-P5CDH antibodies. Polyclonal rabbit anti-P5CDH antibodies were used on Western blot assays to analyze total protein extracts from flowers or leaves of wild type (wt) or p5cdh mutant plants. Samples were loaded on 10% SDS-PAGE gels and analyzed with anti-P5CDH (1/300) and secondary goat anti-rabbit (1/20000) antibodies. Membranes were scanned with Odyssey Infrared Imaging System (LI-COR Biosciences) for detection of secondary antibody (green) and RuBisCo (red). Merge of both channels is shown at the bottom. LSR: large subunit of RuBisCo. [file 1471-2229-14-21-S2.tiff]

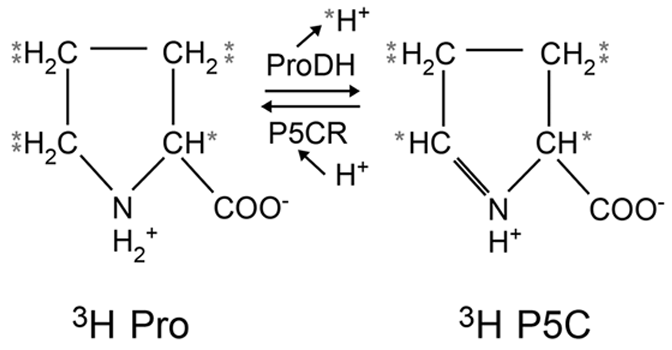

Supplement: Additional file 3 — Radiolabelled 3 H-Pro molecule used as substrate for quantification of ProDH activity in vitro, and its transformation into P5C. [file 1471-2229-14-21-S3.tiff]

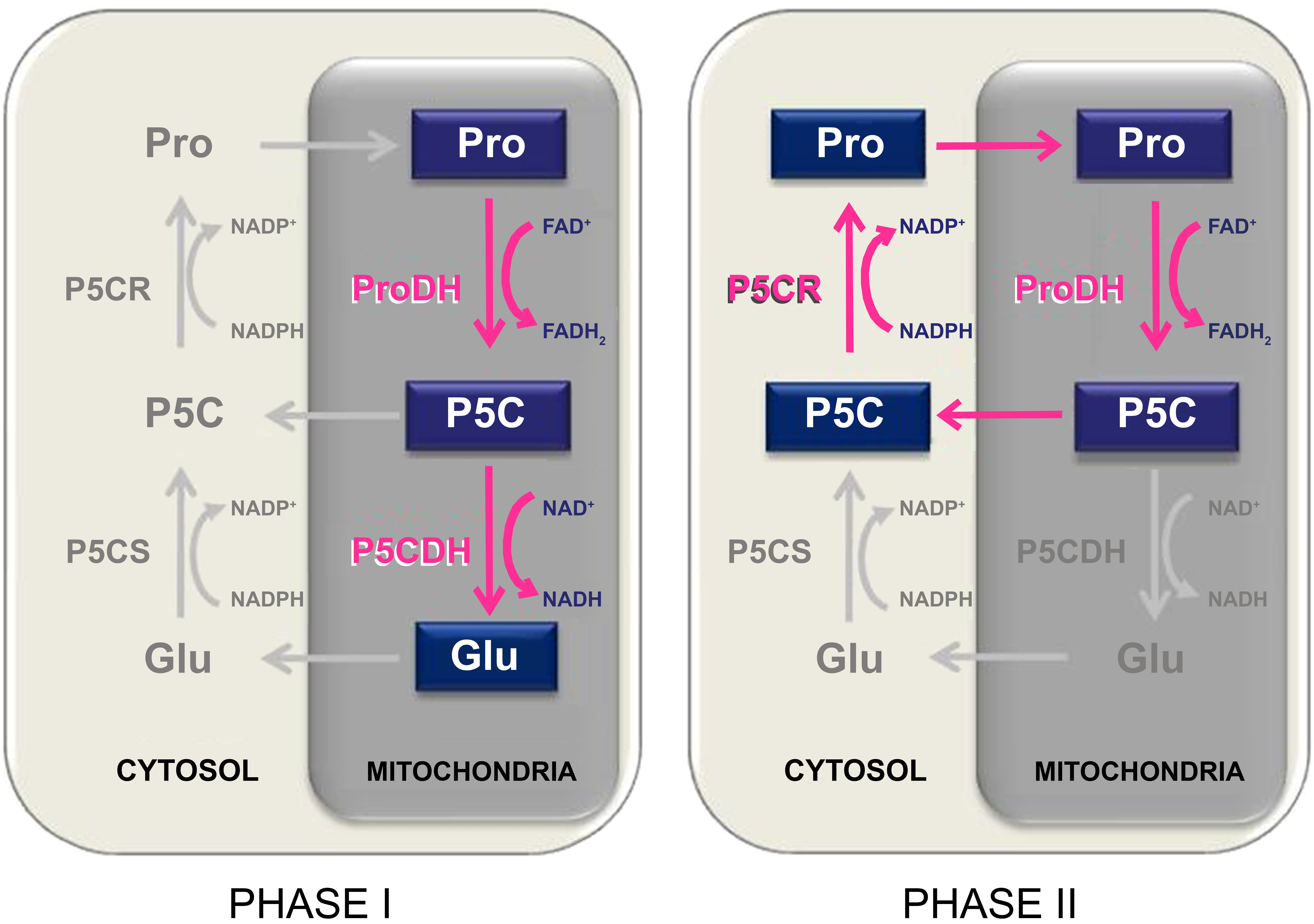

Supplement: Additional file 4 — Presumable action of ProDH in phases I and II of HR. The results of this study suggest that ProDH acts in two different metabolic contexts throughout HR. Prior to oxidative stress (phase I) the enzyme likely acts together with P5CDH producing complete Pro oxidation. These enzymes may somehow become uncoupled at the stage of oxidative stress (phase II), where ProDH remains active but does not contribute to Pro consumption or P5C accumulation. Transcriptional activation of P5CR suggests a coupling of ProDH and P5CR at this second phase with consequent stimulation of the Pro/P5C cycle. [file 1471-2229-14-21-S4.tiff]
