# Supplementary material for: Context of action of Proline Dehydrogenase (ProDH) in the Hypersensitive Response of Arabidopsis
Source: BMC Plant Biol. 2014 Jan 13;14:21. doi: 10.1186/1471-2229-14-21 (PMC3902764; doi:10.1186/1471-2229-14-21)
Supplement: Additional file 5 — Primers and conditions used for sqRT-PCR experiments. # Melting temperature used for each pair of primers. *For each pair of primers the optimal cycle number was selected from a linear amplification range using the following conditions: 1 μg RNA, 500 ŋg random hexamer primers, 200 U M-MLV reverse transcriptase for cDNA synthesis; 1 μl cDNA, 10 μM primer, 25 μl final volume for PCR. The GapC gene, insensitive to Pst treatment (Arabidopsis eFP Browser), was used as internal control. [file 1471-2229-14-21-S5.docx]

**Additional file 5. Primers and conditions used for sqRT-PCR experiments**

| **Gene**  **(AGI Nº)** | **Primers** | **Tm#**  **(ºC)** | **Cycles*** |
| --- | --- | --- | --- |
| *ProDH1 (At3g30775)* | Fw: 5´-TGATGGAGAAAGCATCAAACGG-3´ | 60 | 31 |
|  | Rv: 5´-TCTCCTCTTAAGTTCCATCCTC-3´ |  |  |
| *P5CDH (At5g62530)* | Fw: 5´-ATGTTGGAGCACATGG-3´ | 60 | 28 |
|  | Rv: 5´-GTGACGAGTTCGTAGG-3´ |  |  |
| *P5CS2 (At3g55610)* | Fw: 5´-CCAATATTCTCCACGTCCGCTTCTTC-3´ | 60 | 26 |
|  | Rv: 5´-GATCTCCGTCATAGTTTGTGTCTATC-3´ |  |  |
| *GapC (At3g04120)* | Fw: 5´-CACTTGAAGGGTGGTGCCAAG-3´ | 60 | 25 |
|  | Rv: 5´-CCTGTTGTCGCCAACGAAGTC-3´ |  |  |
| *UBQ5* | Fw: 5´-GTGGTGCTAAGAAGAGGAAGA-3´ | 60 | 25 |
| *(At3g62250)* | Rv: 5´-TCAAGCTTCAACTCCTTCTTT-3´ |  |  |
| *ProDH2* | Fw: 5´-CGTCGAAGCTGCTAAAACCCT-3´ | 60 | 29 |
| *(At5g38710)* | Rv: 5´-CGTTCGATTCTTGACATCTAAG-3´ |  |  |
| *P5CR* | Fw: 5´-CACAGACCGTTCTTGGAGCTG-3´ | 62 | 25 |
| *(At5g14800)* | Rv: 5´-GTGTTGCCCGGAAAGAGCCTTT-3´ |  |  |

# Melting temperature used for each pair of primers.

* For each pair of primers the optimal cycle number was selected from a linear amplification range using the following conditions: 1 μg RNA, 500 ŋg random hexamer primers, 200 U M-MLV reverse transcriptase for cDNA synthesis; 1 µl cDNA, 10 µM primer, 25 µl final volume for PCR. The *GapC* and *UBQ5* genes, insensitive to *Pst* treatment (Arabidopsis eFP Browser), were used as internal control.
